# Taking Students on a Strengths Safari: A Multidimensional Pilot Study of School-Based Wellbeing for Young Neurodiverse Children

**DOI:** 10.3390/ijerph18136947

**Published:** 2021-06-29

**Authors:** Lauren H. Naples, Elizabeth D. Tuckwiller

**Affiliations:** 1 Child Study Center, Yale School of Medicine, Yale University, New Haven, CT 06511, USA; 2 Department of Special Education and Disability Studies, Graduate School of Education and Human Development, Foggy Bottom Campus, The George Washington University, Washington, DC 20052, USA; btuckwiller@gwu.edu

**Keywords:** wellbeing, covitality, mental health, character strengths, social and emotional learning (SEL), executive functioning, neurodiversity, disability, psychoeducation, intervention

## Abstract

There is a robust body of psychological research linking youth mental health and academic achievement. However, students in early childhood are rarely represented in this research, and children with disabilities and/or neurological differences are virtually absent. Thus, the present pilot study explored the effects of a structured psychoeducation program designed to enhance school-based wellbeing (SBWB) for young students who are neurodivergent (ND). This study utilized a quasi-experimental design to investigate the effects of the *Student Strengths Safari* intervention on (1) students’ self-reported covitality and (2) teacher-rated executive functioning to (3) examine data for evidence of a dual-factor model of SBWB. Two classrooms in a suburban, Mid-Atlantic private school were randomly assigned to the waitlist control group (*n* = 14) (1st grade) and the intervention group (*n* = 10) (2nd grade), and quantitative data were analyzed at pretest and posttest to determine intervention outcomes. Key findings produced evidence to support (a) a statistically significant interaction effect for improvements in executive functioning relative to the waitlist control group (*p* = 0.011), and (b) the utility of a new theoretical dual-factor model to advance SBWB for ND students in early elementary education.

## 1. Introduction

Despite robust research evidence linking youth mental health and academic outcomes [1], the limited inclusion of young children, specifically those who are neurodivergent (ND), in this body of research represents a significant gap in the literature. The present pilot study synthesized and applied research from the fields of positive psychology, disability studies, and educational psychology to address this gap. We review seminal literature on the construct of subjective wellbeing (SWB) and research investigating its application in promoting positive youth outcomes and extend these approaches in the present study to support students through the lens of the neurodiversity framework.

The construct of SWB represents an individual’s subjective cognitive (e.g., life satisfaction) and affective (e.g., happiness) evaluations of their personal life experiences [2]. Life satisfaction has been defined as the cognitive appraisal of one’s life, and happiness has been conceptualized as the perception (or higher frequency, when measured) of positive emotional experiences relative to negative experiences [2,3]. This definition has been regarded as just one factor in the overarching model of SWB representing the traditional hedonic view of mental health, focused specifically on emotional wellbeing [4]. Efforts to capture a more holistic definition of SWB led to the integration of eudaimonic wellbeing [5], regarded as the influence of optimal individual and societal functioning into conceptualizations of SWB. Subsequently, social wellbeing and psychological wellbeing emerged from the eudaimonic tradition as influential to overall SWB.

Higher levels of SWB have been causally linked to occupational success, positive mental and physical health, and satisfying interpersonal relationships [6]. Further, individuals who have reported higher SWB have also demonstrated high levels of positive personality traits, such as cooperation, confidence, creativity, tolerance, and altruism [6,7]. Therefore, a systematic focus on improving SWB is a promising endeavor to facilitate positive developmental trajectories across multiple domains. 

### 1.1. The Dual-Factor Model of Mental Health

High levels of SWB have been shown to mitigate the negative effects of psychological distress and other adversities, for both adults and youth [8,9]. A compelling body of research has emerged around the role of SWB in individuals’ experiences of mental health, resulting in a holistic conceptualization referred to as the dual-factor model of mental health [10]. Unlike the traditional model of mental health, which presumes lack of psychopathology (PTH) is equivalent to positive SWB, the dual-factor model of mental health conceptualizes mental health as both the absence of PTH and the presence of factors, traits, and dispositions associated with SWB [10,11]. The recognition that SWB and PTH are distinct, yet interrelated constructs has prompted significant focus on positive indicators as critical to complete mental health (CMH) assessment. This framework has given rise to the prominence of SWB as a key component in forming a comprehensive view of mental health that is necessary to promote optimal functioning [10].

Seminal research on large-scale national surveys with adults provided evidence to suggest that CMH—higher levels of SWB, in conjunction with low levels of PTH—were associated with more favorable occupational, social, physical, and psychological outcomes when compared to outcomes of adults who reported lower levels of SWB in the absence of PTH [4,12]. Findings associated with studies of these distinct group classifications have provided evidence for the utility of a dual-factor model of mental health in which the underlying theoretical constructs of mental health and mental illness differ in terms of overall mental wellbeing [13]. For example, Keyes’s model of flourishing mental health (2005) represents a dual-continuum approach to mental health that has been used in conjunction with the wellness diagnostic criteria from the DSM-IV [14] to classify individuals as flourishing (i.e., experiencing the presence of mental health), languishing (i.e., experiencing the absence of mental health), or moderately mentally healthy [4,12].

### 1.2. The Dual-Factor Model of Mental Health for Children and Adolescents

The dual-factor model of mental health has also been substantiated in research with children, adolescents and young adults, demonstrating associations between higher SWB and positive life factors, beyond mitigating PTH. Findings from studies with children [10] adolescents [11,15,16], and young adults [17,18] indicate that higher levels of SWB are positively related to academic achievement, academic motivation, school engagement, positive peer and teacher relationships, physical health, and self-esteem. However, despite empirical evidence that SWB supports long-term psychological and cognitive health, successful relationships, and improved academic outcomes, this construct has remained largely unexplored in early childhood education and disability studies [6,7]. 

In the present study, we adapted the dual factor model of mental health to explore school-based wellbeing (SBWB) using the Primary model of covitality [19]. This expanded conceptualization elevates a strengths-focused orientation that is universally valuable in the promotion of improved mental health and wellbeing [20].

### 1.3. Dual-Factor Approaches for Children with Disabilities

A strengths-based focus may be meaningful, particularly for the field of special education, and especially in early childhood special education. Early childhood intervention and special education programs in the United States serve children from birth to 8 years old who have or are at risk for developmental delays/disabilities or other special needs [21] (note: we prefer and use the less evaluative phrasing of “neurodiverse/neurodiversity” or “neurodivergent/neurodivergence (ND)” to reflect our disability studies lens). The neurodiversity paradigm advances the position that neurological differences result from normal, natural variation in the human genome [22] and rejects the longstanding tradition which views neurodivergence through the lens of PTH. Young children who have been identified, formally or informally, as ND often experience additional challenges in school and life as a result of omnipresent obstacles embedded in neurotypical-normed settings that situate their differences as undesirable and in need of intervention. Asset-focused approaches build upon strengths inherent to individuals, rather than solely “intervening” to address individuals’ “weaknesses” and “deficits.” The implementation of these strength-based approaches can both reduce the likelihood of self-perceived hopelessness and helplessness resulting from repeated deficit-focused experiences, and serve as a protective factor, mediating the negative effects of psychological distress and/or the challenges that can arise for ND students. When translated and applied to a school setting, this dual-factor model requires a holistic approach to intervention that does not merely rely on remediation of observed “weaknesses” but also considers students’ perspectives of their own wellbeing to empower them to build upon their strengths and assets as active agents throughout their course of development.

Considering the salience of SWB for student outcomes, and the typical exclusion of young ND students in mental health research, there is an urgent need to extend the conceptual model of SWB to be inclusive of all students to promote comprehensive understanding of factors associated with optimal outcomes for every child. Further, conceptually relevant intervention studies and appropriate progress monitoring of their effects within the school context must be explored to determine how best to promote SWB within this expanded framework [11,16].

#### Asset-Based Approaches

Historically, the concept of disability has been situated in a medicalized deficits-focused framework [23]. For decades, youth have been exposed to practices aimed at remediating their perceived deficits, from applied behavior analysis [24] and social skills treatments [25] to the extreme Scared Straight program, in which youth considered “at risk” are exposed to prison life to deter them from risky behavior [26]. For many children, school experiences highlight their perceived intrinsic deficits, and prioritize efforts to remediate these deficits, often to the exclusion of their inherent strengths. These approaches not only undermine a child’s self-perceptions and confidence [27], but also hinder educational research and practice, leading to constrained development of theory and educational methods [23], and blind spots in our philosophical and theoretical understandings of the phenomenon of disability [28].

Fortunately, the conceptualization of disability is evolving from a deficits-based framework to one rooted in a socio-ecological framework, driven by a person-fit model, through which optimal functioning is dependent on the interactions of individuals’ capabilities and their environmental demands [29]. This framework facilitates the integration of positive psychology through a strengths-based approach to special education by justifying a focus on recognizing and fostering strengths of individuals while concurrently ameliorating environmental factors that act as barriers to growth and functioning. 

The field of positive psychology suggests that universally applicable, multitarget strengths-based programs that integrate assessment and positive intervention into routine educational practice with all students [30] can systematically develop “positive feelings, positive behaviors, or positive cognitions’’ [31] (p. 467) and buffer against PTH [32]. Recent studies have linked universal positive psychology interventions to sustained improvements in students’ subjective wellbeing, class cohesion, and learning engagement [33,34,35], and single-target PPIs have been shown to improve elementary-aged students’ positive affect, class cohesion, engagement, and self-esteem [33,36].

Niemiec and colleagues [37] identified a critical need for merging the fields of positive psychology and disability studies by introducing and modifying character strengths assessment and intervention into work supporting individuals with intellectual and developmental disabilities. This goal may be accomplished by leveraging the science of character, which is derived from the mission of positive psychology to understand and develop strengths, often referred to as virtues, through the study of human flourishing [38]. The notion of character strengths—the core psychological processes that define and drive human virtues—emerged from this work [39]. Moreover, Armstrong and colleagues [40] likewise emphasized the importance of assessing and building upon character strengths associated with early childhood development, adaptation, and well-being, lending further support to the call for an interdisciplinary merger to optimize student outcomes for young children. 

### 1.4. VIA Classification of Strengths

Character strengths represent positive traits that every individual possesses to guide thinking, feeling, and behaviors which are beneficial to promote both a flourishing individual and society [41]. They have become a significant focus in the field of positive psychology as the building blocks for cultivating psychological orientations that promote optimal life outcomes [37,39]. The VIA Classification of Strengths was crafted in direct contrast to the Diagnostic and Statistical Manual of Mental Disorders [14] and serves as a comprehensive manual of 24 character strengths inherent to all individuals [39]. These character strengths are organized according to six higher order virtues—wisdom, courage, humanity, justice, temperance, and transcendence—which have been empirically validated as culturally and historically relevant across diverse contexts [42,43]. This classification system provides a common language for researchers and practitioners and serves as a foundational point of reference for measuring and building upon these virtues, which correlate to overall SWB.

### 1.5. Character Strengths in Early Childhood

Significant strides have been made in recent years to define and measure SWB and corresponding character strengths in young children. Decades of prior research have steadily built the case that social-emotional health, prosocial skills, academic outcomes, and SWB are interrelated. Early research linking children’s self-awareness of the connection between their peer relationships and their school adjustment [44,45] gave way to subsequent research linking prosocial skills to learning-related skills (i.e., self-regulation, on-task behavior) [46]. Later, longitudinal evidence indicated that kindergarten learning skills were related to school success through second grade, but the relationship was significantly mediated by self-regulation skills [47]. These early findings suggested a connection between skills related to character strengths and positive school outcomes.

The first and only study to investigate character strengths in very young to early elementary-aged children (3–9 years-old) analyzed open-ended parental responses to questions about their children’s character strengths and happiness [48]. All 24 VIA character strengths were represented in parent reports, which suggested that character strengths may be linked to developmental stages across the lifespan. For example, hope and zest were more commonly reported among youth than in older populations, whereas cerebral strengths, such as appreciation of beauty, tended to emerge later in life [48,49]. Park and Peterson [48] confirmed character strengths begin to emerge as early as the age of 1 year, and consistent groups of character strengths can be recognized as early as the age of 3 years [50]. Findings such as these provide a compelling rationale for exploring the development and cultivation of character strengths at an early age [48,49]. Furthermore, Park and Peterson [48] emphasized the necessity of multitarget—addressing two or more psychological dispositions related to SWB—and multimodal assessment and intervention processes, which consider objective informants, structured surveys, and other measures for young ND children, and/or children with disabilities, as these groups have been overlooked in prior research.

### 1.6. Covitality

Weiss, King, and Enns [51] first introduced the concept of covitality as coexisting traits in a study examining dominance in chimpanzees. They defined covitality as “phenotypic or genetic correlations among positive traits such as wellbeing, confidence, and health” [51] (p. 1147). This seminal research laid the groundwork for positive psychology researchers to identify groups of coexisting human personality traits linked to psychological constructs that, when measured as a higher order construct, account for greater variance in outcomes than the additive effect of the individual constructs alone [52]. In short, this concept is predicated on the notion that combined groups of traits potentiate the effects of one another to a greater magnitude than the individual relative contribution of each factor. Most simply, the suggestion is that the value of the whole is greater than the sum of its parts. Rather than conceptualizing internal assets in silos, through individual measurement and narrow practical implications, covitality reflects the complexity of groups of traits and the potentiation of their effects when they coexist. This orientation to understanding groups of traits that support optimization of psychosocial outcomes is not only more authentically representative of the human experience, but also reflects the socioecological nature of school contexts. 

The social ecology around a developing child and the interpersonal interactions that occur therein significantly shape learning and development. Such experiences do not occur in a vacuum; rather, they result from constant cognitive integration of and emotional adaptation to those experiences. Recent research emphasizes the potential for capitalizing on opportunities to enhance covitality, or “the synergistic experience of wellbeing that results from the interactions of multiple school-grounded positive traits in youth” [19] (p. 758) by targeting coexisting character strengths linked to optimal student outcomes. In adolescents, covitality is positively correlated with SWB [53,54], and further evidence identifies covitality as a unique predictor of SWB [55]. Results from the first empirical investigation of student covitality with elementary-aged students established its significance as a higher order construct that more accurately predicted prosocial behavior, caring relationships, school acceptance, and school rejection than did individual measurement of the underlying psychological dispositions associated with SWB [19]. 

A few multitarget interventions have addressed student covitality constructs by incorporating evidence-based practices into daily programming for students in middle and elementary school [35,56,57,58,59,60,61]. For example, the Well-being Promotion Program [35,56,59,60,61] is a comprehensive multitarget, multicomponent (e.g., teachers, parents) initiative that has been implemented in modified formats for student classrooms as early as 3rd–5th grade [35,56,61]. However, the present study is the first to investigate direct application of the covitality model through intervention with early elementary students (i.e., 1st and 2nd grade), as well as the first to investigate its effects for ND children. 

### 1.7. The Present Study

Evidence for the utility of a dual-factor model of mental health has been established sufficiently for older students within school contexts, but this approach has not been translated to assessment or intervention application in early childhood settings, nor for students at-risk and/or those who are ND. The dual-factor model of mental health provided sound conceptual framing to explore the development and implementation of a pilot covitality intervention, centering strengths-based development, for young elementary-aged ND students and associated challenges, particularly in executive functioning. Given the evidence indicating the importance of covitality for SBWB, the 8-session intervention was designed to expand upon comprehensive models of youth social-emotional strengths by offering structured, school-based experiences to increase students’ awareness of critical personal assets and provide opportunities to explicitly develop these assets in a school environment. Although the pilot intervention incorporated perspectives from related yet distinct fields (e.g., social-emotional learning; positive psychology; educational psychology, special education), given the existing literature indicating the importance of covitality to SBWB, we targeted the four first-order psychological factors that comprise student covitality at the primary level—school gratitude, student zest, school optimism, and student persistence [19]—as the core of the intervention. During regular classroom time, over a four-week period, students participated in an age-appropriate “Strengths Safari” (twice weekly; 30-min per lesson). They had opportunities to learn about, reflect upon, and build these four personal assets during classroom activities and via short, supplemental activities (“Cheetah Challenges”) completed between lessons. 

The study reported here represents Study 1 embedded within a larger school-situated, design-based research project conducted with a research partnership school over the course of 1 year, in fulfillment of the first author’s dissertation milestone [62]. For Study 1, we investigated the following research questions:

1. Do students who participate in an 8-session covitality intervention demonstrate improvements in self-reported covitality from pretest to posttest?
2. Do students who participate in an 8-session covitality intervention demonstrate improvements in teacher-rated executive functioning compared to their waitlist control group peers?
3. Do multidimensional student profiles, constructed from established BRIEF-2 *T* score clinical descriptors [63] and SEHS-P strengths classification thresholds [64], indicate a practically meaningful dual-factor model of school-based wellbeing for young ND children?

## 2. Materials and Methods

Over the course of the year-long design-based research project, we implemented an advanced mixed methods research (MMR) design in which a convergent core, formed by qualitative and quantitative data, was embedded within an overarching quantitative quasi-experimental framework (QUAN + qual) [65] to broadly explore SBWB for young ND students. Here, we detail Study 1, the quasi-experimental intervention component, embedded within this larger research project. For Study 1, we used quantitative methods to measure intervention outcomes by examining mean differences over time in teacher-rated executive functioning (EF) (BRIEF-2) [63] and student-rated covitality (SEHS-P) [19] between students in the intervention and waitlist control groups. We also developed multidimensional student profiles to examine the data for evidence of a dual-factor model of SBWB.

### 2.1. Research Context 

This study was conducted in partnership with a non-profit, nonpublic, suburban school serving neurodiverse students in the Mid-Atlantic region of the United States. The U.S. education system is comprised of public schools, which receive government funding (e.g., traditional public schools, public charter schools); nonpublic schools, not primarily supported by government funds (e.g., independent schools, private schools); and homeschools. Although the federal government provides policy guidance via federal education legislation, each state maintains the primary responsibility for developing and implementing state-specific education policies and practices in compliance with those guidelines. Local education agencies (e.g., districts) within states also have some flexibility in implementing state-mandated education policies. Nonpublic schools, whether parochial or not religiously affiliated, have access to some government support, coordinated through their state’s Department of Education, including information, advocacy, selected funding (e.g., Title I funding to improve academic achievement for learners who are economically disadvantaged), and services (e.g., programming for drug-free schools and communities) [66]. Nonpublic schools have more flexibility in delivery models, curricula, service provision, and personalization of education compared to traditional public schools. Nearly 5 million students are enrolled in nonpublic schools in the United States [67].

Our nonpublic research partnership school for this study takes a unique approach to educating neurodiverse learners through universal implementation of a specially tailored social learning curriculum. Additionally, the school context is rooted in a history and collective efficacy of research-to-practice program evaluation efforts and public dissemination of findings. Faculty and staff at this site present at national conferences and publish research results in peer-reviewed journals. Thus, the expertise and interests of the staff, and the operational nature of the school, provided a robust environment in which to explore a dual-factor model of SBWB for ND students through structured opportunities to develop, evaluate, and infuse strengths-based intervention activities into the established social learning curriculum. Additionally, the small size of the school supported targeted investigation of classroom-specific intervention outcomes.

### 2.2. Sampling Design

The research process was co-designed with a local research partnership school and tested within this authentic educational context using convenience sampling. The intervention sample was nested within the larger school population (*N* = 45, pre-K to 2nd grade), and the 1st- and 2nd-grade student classrooms were randomly assigned to the intervention or waitlist control group. The waitlist control group continued to receive instruction as usual while the intervention group participated in the pilot covitality intervention. This purposeful approach to study sampling was especially pertinent to the design and testing of intervention strategies which have not yet been utilized with early elementary students or participants who experience neurological differences that impact learning and social cognition. Thus, the intervention program was piloted in 1st and 2nd grade to provide baseline data as the foundation for future adaptation and expansion.

### 2.3. Student Participants

As this study was conducted at a site uniquely designed to meet the needs of young ND children who experience a range of developmental differences, students in this sample represented a heterogeneous group of neurodiverse learners. At the time of this study, a total of 24 students were enrolled in grades 1–2 and comprised the participant sample, with 14 students in 1st grade (10 boys, 4 girls) and 10 students in 2nd grade (9 boys, 1 girl). Due to the early age rage (*M* = 6.0 years) and developmental level of the participants, valid disability diagnostic data were inconsistent and difficult to obtain for each student. In lieu of formal disability diagnoses, teachers provided insight from their own observations to describe participant ND characteristics, identifying varied and persistent behaviors across the student sample that were symptomatic of autism, attention deficit/hyperactivity disorder (ADHD), communication impairments such as expressive/receptive delays and pragmatic language disorder, anxiety, emotion regulation challenges, and fine/gross motor difficulties. 

### 2.4. Quantitative Measures

#### 2.4.1. Covitality

Students’ levels of covitality were measured by participant responses on the SEHS-P [19]. The SEHS-P [19] is a self-report strengths-based instrument designed to measure positive psychological functioning in school. This 16-item scale comprises four subscales (school gratitude, student zest, school optimism, and student persistence) to assess positive school-grounded traits in youth that are linked to student wellbeing and school engagement. The subscales measure an individual’s perceptions about what they think, feel, and do at school. Cumulative subscale scores of the four first order factors reflect an overall composite score of student covitality, or the synergistic experience of wellbeing that results from interactions among cumulative school-grounded traits [19]. Higher scores reflected higher levels of covitality. The SEHS-P [19] has established full factorial invariance across genders and good internal reliability across subscales: school gratitude (α = 0.71), student zest (α = 0.78), school optimism (α = 0.71), student persistence (α = 0.80), covitality (α = 0.89), and prosocial behavior (α = 0.81) for students in grades 4–8 (age, *M* = 11.1 years) [19,52,68,69]. The survey is free to download, and the author granted permission for use in the present study.

#### 2.4.2. Executive Functioning

Students’ levels of executive functioning (EF) were measured by teacher ratings on the BRIEF-2 [63]. This assessment is an appropriate measure of EF for very young students (e.g., age 5–18 years) with developmental and acquired neurological variations such as learning disabilities, ADHD, traumatic brain injury, low birth weight, Tourette’s Disorder, and autism. The scale yields a clinical global composite score which encompasses three subscales measuring student regulation in the domains of behavior (i.e., inhibit, self-monitor), emotion (i.e., shift, emotional control), and cognition (i.e., initiate, working memory, plan/organize, task-monitor, material organization); inconsistency, negativity and infrequency scales provide additional validity information. As a third-party measure of executive functioning, the BRIEF-2 [63] is used commonly for diagnosing forms of neurodivergence and tracking student progress for children who experience behavioral, emotional, or cognitive distress in school.

The internal structure of the BRIEF-2 indicates adequate validity; item-total correlations revealed moderate to strong membership for each scale on the teacher form (coefficients range from 0.50 to 0.83) [70]. Moderate to strong correlations between other measures of behavior and cognition with the BRIEF-2 [63] suggest adequate concurrent validity. In addition, beyond established reliability and validity, the evaluation is efficient, requiring only 10 min to complete. The BRIEF-2 [63] demonstrates high internal consistency on all index scores on all forms (e.g., parent, teacher, self). Reliability coefficients for the teacher form, utilized in this study, range between 0.88 and 0.98, with index and composite scores ranging between 0.94 and 0.98 [70]. However, interrater reliability coefficients for teacher-teacher pairs range between 0.42 and 0.70 [70]. Thus, it was important for us to have one rater complete the BRIEF-2 assessments over the course of the study. The school curriculum coordinator served as the study evaluator of students’ executive functioning. Her established rapport with student study participants facilitated sufficient familiarity to yield accurate pretest and posttest evaluations. The curriculum coordinator was not otherwise involved in study implementation beyond assisting with posttest SEHS-P [19] collection for one student in 1st grade. Purchase of the BRIEF-2 [63] was required for use and the 63-item teacher core form was completed manually by the school’s curriculum coordinator for all students in 1st and 2nd grade at pretest and posttest.

### 2.5. Data Collection Procedures

#### 2.5.1. Pretest Data Collection

Pretest data were collected during the third week of the school year to allow time for students to become acclimated to the school environment, and for teachers to become familiar with their students. The first author collected quantitative covitality data over the course of one day. The BRIEF-2 [63] was provided to the school curriculum coordinator to facilitate pretest EF assessment the week following the collection of covitality data, on the first day of the intervention. All participating students were present on the day of pretest data collection.

The SEHS-P [19] has not been used in prior research with ND students in early childhood education; therefore, it was modified for developmentally appropriate administration. Adaptation occurred in consultation with a team of practitioners who were knowledgeable of students’ comprehension and language skills to modify the survey questions to a pre-K comprehension level prior to administration. Language modifications were minimal, and adaptations included visual cues and developmentally appropriate administration strategies informed by suggestions outlined to supplement the VIA-Youth [71] survey with adolescents (aged 10–17 years) with intellectual and developmental disabilities.

Students in 1st grade sat at desks arranged in a U-shape around the classroom with two co-teachers and the first author. The student group exhibited some challenging behaviors; for example, intermittent outbursts triggered sensory issues among a few students, while other students were resistant to completing the survey and wanted to finish the activity quickly. Thus, data were collected in 1st grade over the course of two sessions, as time constraints did not allow for complete collection within one session. The second session occurred following data collection in Grade 2 and incorporated adaptations that evolved and were integrated throughout the day in other classrooms to streamline the process. Students in 2nd grade were arranged in small groups, with 10 students divided among three tables. Two co-teachers and the first author facilitated the SEHS-P [19] survey completion.

#### 2.5.2. Posttest Data Collection

Posttest data collection occurred one week following the completion of the intervention through repeated quantitative measures of student covitality and executive functioning. All students in 1st and 2nd grade provided responses to the SEHS-P [19] on the same day, and the curriculum coordinator completed the BRIEF-2 [63] for all students the following week.

### 2.6. Intervention Implementation

Comprehensive models of youth social-emotional health have indicated that beneficial character strengths, dispositions, and personal assets can promote positive experiences and outcomes in childhood and throughout the lifespan [20]. The Student Strengths Safari [62] was developed to provide structured school-based psychoeducation centered on four personal assets that elevate student covitality through implementation of eight sequenced activities targeting (a) gratitude, with journaling and a modified gratitude visit; (b) optimism, with positive reframing to foster a growth mindset and envisioning one’s “best student self;” (c) persistence, with identifying concrete steps to achieve “best self” goals and using self-talk strategies to overcome barriers; and (d) zest, with practicing mindfulness during a nature walk and doing a “student skills scavenger hunt” to promote positive peer relationships (see Appendix A Program Overview; contact the first author for additional information). Although these factors do not represent an exhaustive account of all youth-oriented strengths, the evidence supporting their importance in school-based experiences of wellbeing indicated their potential as a robust research-based starting point for the pilot intervention. Beyond providing explicit exposure and instruction to young children in the “what” of these factors, psychoeducational approaches also empower youth with information about “how” to reflect upon and build personal strengths over the lifespan. Thus, the Student Strengths Safari was designed to educate young ND children about these strengths, as well as encourage them to be active agents in the development of complete mental health in particular, and cognizant of their strengths and capabilities to build personal assets in general.

Logistical procedures for intervention implementation were developed in consultation with the school director during a 6-month study design phase. Prior to implementation, the intervention curriculum was introduced to classroom teachers during their professional development week that occurred at the end of August and was presented to parents at “Back-to-School Night” in mid-September. 

The brief intervention was implemented in a sequenced format of eight 30-min sessions, delivered twice weekly for 4 weeks during the regular school day. The sessions were conducted primarily by the first author, with assistance and feedback from the two classroom teachers before, during, and after each session. Ongoing communication and cooperation allowed us to collaborate and respond to what worked, what did not work, and what could be improved, adapted, or linked with other classroom lessons to enhance learning experiences. 

Each intervention session built upon previous lessons and included activity prompts for students to practice skill-building outside of structured sessions and subsequently share reflections in their next meeting. When challenges emerged, for example, around limitations on time that necessitated restructuring sessions to accommodate student absences and the provision of sufficient time for skill-building between sessions, we were able to address these issues in real time with flexible planning and instructional support from classroom teachers.

#### Adaptation of Intervention Procedures

The first author implemented each session in collaboration with teachers to integrate adapted strategies as students and teachers alike progressed through the program. Following each session, the first author collected student workbooks and redistributed them at the beginning of the next session so that activities initially planned for each session could be revised for accessibility based on teacher feedback, and the updated materials could be inserted into the student workbooks. Classroom teachers used their expertise of the classroom culture and knowledge of individual students’ learning needs to explain activity instructions, clarify questions, and manage behavior through language that was both developmentally appropriate and reflective of the school-wide integrated social learning curriculum. 

Further, teachers linked the intervention session activities and themes to students’ academic activities to help with lesson comprehension, transferability, and generalizability. Teachers were instrumental in adapting activities for efficiency and efficacy by providing student- and group-specific assistance in the moment and offering suggestions for future sessions. 

In keeping with the strengths-focused theme of the psychoeducation curriculum, teachers also provided suggestions for proactive adaptations that would minimize the burden on students seeking help. For instance, to prepare for certain activities prior to the session, teachers rearranged the students’ desk pods to accommodate similar learning needs in small groups. This way, one teacher was stationed at each of the three pods to facilitate completion of the workbook activity among small student groups who thrive with similar accommodations.

Moreover, teachers suggested different methods to prepare session materials in advance of each lesson to reduce the number of writing-intensive activities that required unnecessary effort from students. In sum, collaboration with and contributions from teachers and administrators were invaluable throughout the intervention process, including scheduling, implementation, and adaptation to ensure the curriculum was accessible for all students.

### 2.7. Data Analysis

Quantitative data were inspected visually and analyzed through descriptive and inferential statistics using IBM Statistical Package for the Social Sciences (SPSS Statistics, Version 25, IBM, Armonk, NY, USA). To test the data for normality and homogeneity of variance, we used the Shapiro–Wilk test and Levene’s test. Levene’s test indicated homogeneity of variance for all executive functioning subscales and the global composite EF score, as well as covitality and all of its first order factors (*p* > 0.05). The Shapiro–Wilk test indicated a normal distribution of EF scores across both groups at both timepoints (*p* > 0.05). Covitality was also normally distributed for both groups at pretest and for the intervention group at posttest (*p* > 0.05). However, covitality scores were not normally distributed for the waitlist control group at posttest (*p* = 0.019); therefore, we provide, yet urge extreme caution in interpreting covitality results parametrically for the waitlist control group at this time point. 

Between- and within-groups differences in mean student scores were calculated using mixed factorial ANOVA to determine univariate intervention effects on self-reported covitality and teacher-rated executive functioning over time, and effect sizes were calculated using partial eta squared. Further, the SEHS-P [19] has been examined concurrently only with similar self-reported construct measures and its predictive utility with other socially valid outcomes, such as executive functioning, warranted investigation [68]. Thus, relationships between covitality and executive functioning were examined utilizing the SEHS-P [19] and BRIEF-2 [63] for evidence of a dual-factor model of SBWB.

### 2.8. Human Participants and Ethical Considerations 

This study was conducted with the full participation of a local partner school, and the pilot intervention was adopted to supplement classroom instruction. In collaboration with the school staff, activities were integrated into the regular school day and program evaluation served to inform ongoing practice. Therefore, implementation of a low risk covitality intervention, co-developed with and adopted by the partner school as standard practice in their educational programming, was found exempt (per federal regulations under category (1) by the institutional review board. Accordingly, the data collection, analysis, and reporting procedures were consistent with the guidelines, institutional policies, and approved practices of the partner school to ensure students’ rights were upheld and maintained to the greatest extent of the law.

## 3. Results

We analyzed intervention outcomes quantitatively using multiple repeated measures to answer the following research questions:

1. Do students who participate in an 8-session covitality intervention demonstrate improvements in self-reported covitality from pretest to posttest? Since the covitality data were not normally distributed at posttest for the waitlist control group, we only interpret within-group differences on scores of covitality at each time point for the intervention group below.
2. Do students who participate in an 8-session covitality intervention demonstrate improvements in teacher-rated executive functioning compared to their waitlist control group peers?
3. Do multidimensional student profiles, constructed from established BRIEF-2 *T* score clinical descriptors [63] and SEHS-P strengths classification thresholds [64], indicate a practically meaningful dual-factor model of school-based wellbeing for young ND children?

### 3.1. Findings

All 24 participants responded fully to the SEHS-P [19] at pretest and posttest. This measure provides subscale scores for each of the four first order factors in the primary model, as well as an aggregate score for the second order factor of covitality. Because the SEHS-P [19] has not been used in prior research with young ND children, we calculated the internal consistency of the second order covitality factor using Cronbach’s alpha for all participants (*n* = 24). The cumulative scale demonstrated excellent reliability (α = 0.908), providing novel evidence of whole-scale reliability of the SEHS-P [19] as a measurement tool to assess covitality in young ND children. 

Additionally, a single rater completed the BRIEF-2 [63] for all participants at both timepoints. The GEC score from the BRIEF-2 [63] provided an overall rating of executive functioning for interpretation, such that higher levels of EF were determined by lower GEC scores, and lower levels of EF were determined by higher GEC scores. Participants’ responses on both measures were analyzed descriptively, and results for each scale and subscale are presented below (see Table 1).

Repeated measures mixed factorial ANOVA was conducted to test univariate effects over time, with EF and covitality as the dependent variables, time (pretest, posttest, measured 4 weeks apart) as the within-groups factor, and quasi-experimental condition (waitlist control, intervention) as the between-groups factor (see Table 2). Tests of within-groups contrasts for teacher-rated EF revealed statistically significant results for a main effect of time across four weeks, *F*(1, 22) = 69.6, *p* < 0.001, ηp^2^ = 0.760, and an interaction effect between time and condition, *F*(1, 22) = 7.79, *p* = 0.011, ηp^2^ = 0.261. However, tests of within-groups contrasts for covitality indicated findings were statistically nonsignificant for a main effect of time, *F*(1, 22) = 4.10, *p* = 0.055, ηp^2^ = 0.157, or interaction effect between time and condition *F*(1, 22) = 0.739, *p* = 0.399, ηp^2^ = 0.033. We urge caution in interpreting the data of within-groups contrasts over time, considering posttest data for the waitlist control group was not normally distributed. However, we decided to report these analyses to provide other scholars with a transparent account of our data, as it is the first time the covitality construct has been measured and analyzed for this demographic group. Findings from between-groups repeated measures ANOVA revealed statistically nonsignificant effects over time for executive functioning, *F*(1, 22) = 3.67, *p* = 0.069, ηp^2^ = 0.143, and covitality, *F*(1, 22) = 0.149, *p* = 0.703, ηp^2^ = 0.007 (see Table 3).

**Figure 1:**
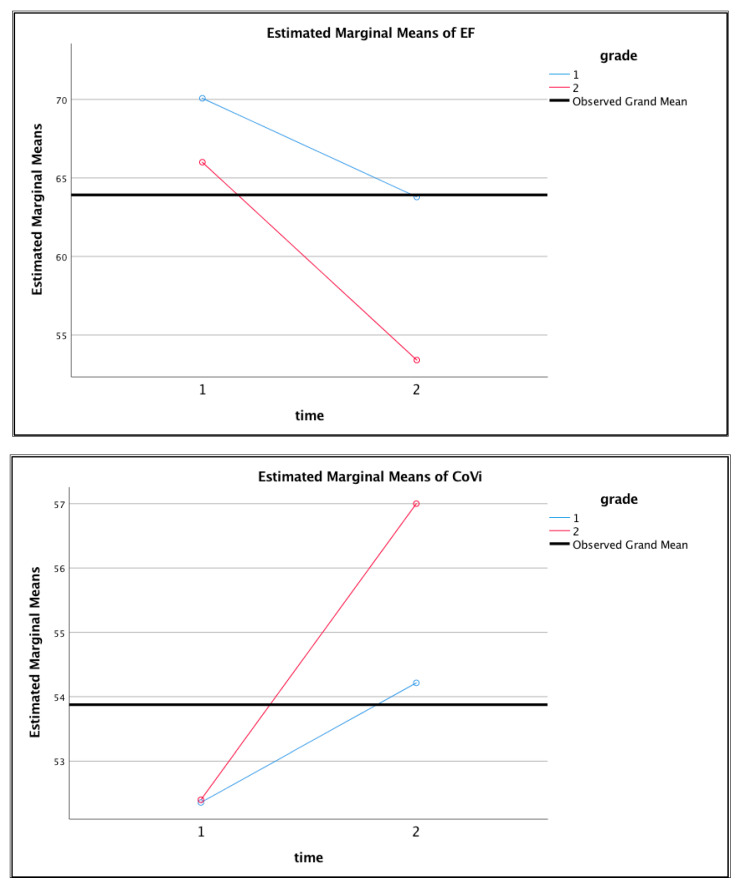
Profile Plots of Estimated Marginal Means. Grade 1 = waitlist control group; Grade 2 = intervention group; EF = executive functioning; CoVi = covitality; Lower ratings on the BRIEF-2 represent higher EF; Higher ratings on the SEHS-P represent higher CoVi.

Subsequently, hypothesis tests were conducted to visually inspect and analyze group differences. Contrast results are reported in a K Matrix (see Table 4), followed by a summary of estimated marginal means (see Table 5) and their corresponding profile plots (see Figure 1). Reduced scores on the BRIEF-2 [63] are favorable and indicate improved executive functioning, whereas higher scores on the SEHS-P [19] are favorable and demonstrate increased covitality over time. Figure 1 illustrates the general trend of improved executive functioning across both groups over time. This trend was an anticipated finding due to the schoolwide integration of social skills instruction as standard educational practice. However, the intervention group showed a more dramatic improvement in executive functioning, supported by a statistically significant interaction effect and correlation coefficient, reported above.

**Figure 2:**
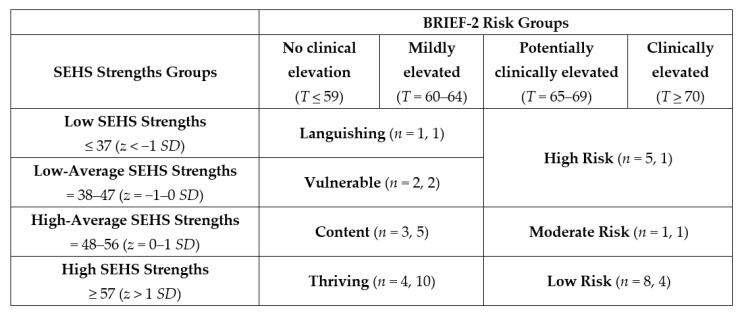
Universal Screening Matrix for School-Based Wellbeing. *N* = 24; *n* = number of students in each risk by strength grouping across 1st- and 2nd-grade classrooms (pretest, posttest). BRIEF-2 = Behavior Rating Inventory of Executive Function^®^, Second Edition [63]; SEHS = Social-Emotional Health Survey [19].

Using previously established thresholds for covitality strengths classification [64] and clinical designations of BRIEF-2 score ranges [63], we inspected student scores on both measures at pretest and posttest and assigned students to categorical profiles aligned with previous literature (see Figure 2). In accordance with the dual-factor model, we were able to assign a category for both a positive indicator (e.g., covitality) and an area of challenge (e.g., elevated EF risk, typically a concern for ND students). The four strengths groups designated students as having high SEHS strengths (z > 1 SD), high-average SEHS strengths (z = 0–1 SD), low-average SEHS strengths (z = −1–0 SD), and low SEHS strengths (z < −1 SD) [64]. The four EF risk groups were identified using the clinical descriptors associated with T scores on the BRIEF-2 [63] to categorize students’ levels of EF risk: no clinical elevation (T score < 60), mildly elevated (T score 60–64), potentially clinically elevated (T score 65–69), and clinically elevated (T score ≥ 70). 

Visual analysis of the ANOVA effects graphs revealed a more dramatic improvement in student covitality which shifted the intervention classroom average scores into the highest student strengths classification group identified in the literature (z > 1 SD), whereas the waitlist control group remained in the same student strengths classification group from pretest to posttest see (Table 6). This finding indicated that although improved covitality was not statistically meaningful, it held practical significance for this group of students. Students in the intervention group demonstrated improvements in teacher-rated EF to such a degree that the observed mean scores for the intervention group indicated no clinical elevation at posttest (see Table 6). This finding is notable, as the waitlist control group also saw improved teacher-rated EF, however, scores were still mildly elevated. These findings provide evidentiary support of a dual-factor model of SBWB in which improved covitality was associated with improved executive functioning.

### 3.2. Multidimensional Student Profiles

**Figure 3:**
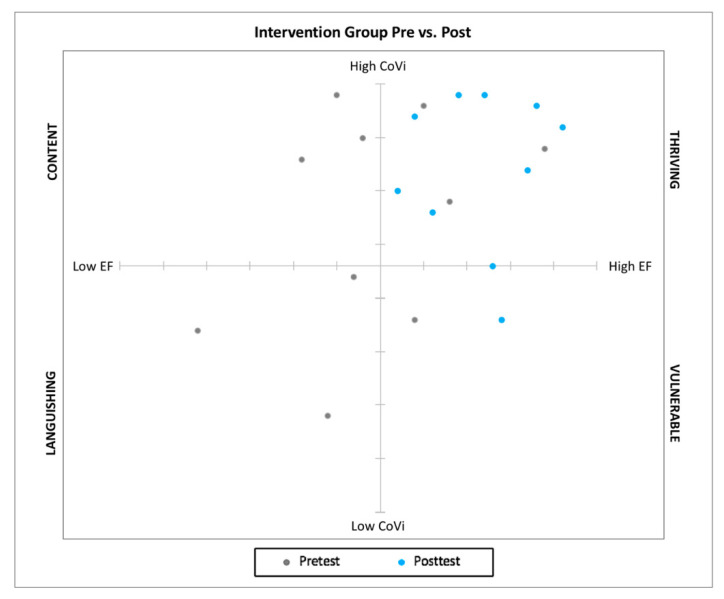
Intervention Group Student Profiles at Pretest and Posttest. EF = executive functioning function; CoVi = student covitality. Map illustrating multidimensional student profiles according to the dual-factor model of school-based wellbeing measured at pretest (gray) and posttest (blue).

**Figure 4:**
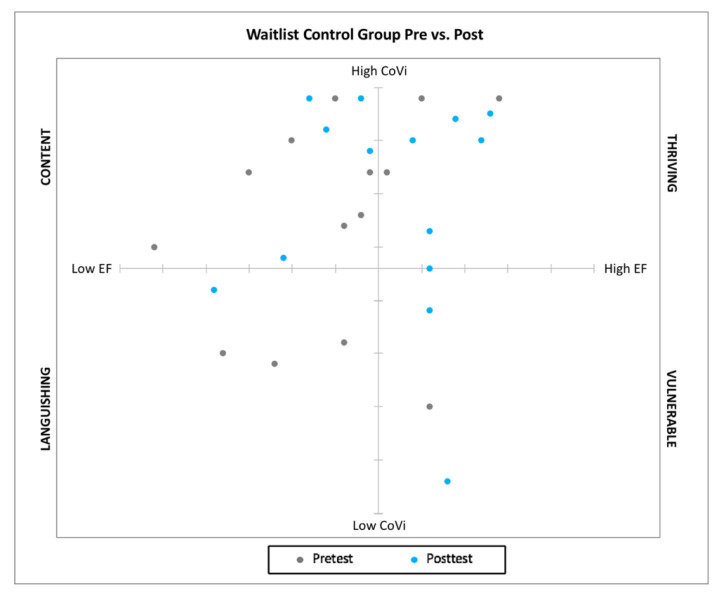
Waitlist Control Group Student Profiles at Pretest and Posttest. EF = executive functioning; CoVi = student covitality. Map illustrating multidimensional student profiles according to the dual-factor model of school-based wellbeing measured at pretest (gray) and posttest (blue).

Building upon the dual-factor model of mental health, in which both positive and negative indicators are conceptualized as important contributors to a holistic understanding of mental health, we considered student-reported levels of covitality and global composite scores of EF for each student to explore potential interactions between these two school-based variables and examine outcomes along dual continua over time (e.g., at pretest and at posttest). Research on the dual-factor model of mental health has indicated that high levels of wellbeing can mitigate the negative effects of psychological distress [15,16,18]. Because young ND students typically struggle with executive functioning, we aimed to explore potential effects of the pilot covitality intervention not only on social-emotional health, but also on observed EF. To visualize young ND students’ profiles from this dual-factor model lens, we plotted students’ quantitative scores from the SEHS-P [19] and the BRIEF-2 [63] onto two axes to create a dual-factor profile for each student on each indicator over time (see Figure 3 and Figure 4). 

Plotting the data in this way facilitated visual examination of outcomes along a dual continuum of skills that are critical to school success. We adapted Keyes’s [12] dual-factor model of mental health profile descriptors (based on high to low SWB and PTH) to current participants’ data from low to high covitality and EF on two axes. We conceptualized the four quadrants as thriving (high covitality (CoVi) and high levels of EF), content (high CoVi, low EF), vulnerable (low CoVi, high EF), and languishing (low CoVi, low EF) (see Table 6 and Figure 3 and Figure 4).

All students within both the intervention and waitlist control groups fell into one of four quadrants on the multidimensional scatter plots at pretest, providing initial evidence of a novel dual-factor conceptualization of SBWB for young ND students. Student profile shifts were expected among both groups as a result of the integrated social skills curriculum implemented at the school. However, the intervention and waitlist control groups differed considerably in their multidimensional profile fluctuations at posttest. As a supplement to the social learning curriculum, the covitality intervention supported dramatic multidimensional profile shifts for the intervention group. For example, 100% of the intervention group with low EF scores at pretest (*n* = 6) shifted to high EF scores at posttest. Additionally, three of the four students who self-reported low covitality scores at pretest shifted to high covitality scores post-intervention, with only one student falling into the vulnerable category, and no students identified as content or languishing at posttest following the covitality intervention (see Figure 3). 

On the other hand, waitlist control group student profiles did not shift nearly as dramatically in either EF or covitality at posttest. Four of the 10 students in the low EF group at pretest shifted to high EF at posttest, and 3 of 4 students who self-reported low covitality at pretest remained in the low covitality group, with only one student shifting to high covitality at posttest. Altogether, less than half of the waitlist control group students were identified as thriving, whereas five students were content, two were vulnerable, and one student was identified as languishing, showing a similar profile pattern from pretest to posttest (see Figure 4).

### 3.3. Summary of Findings

This study investigated effects of a novel pilot covitality intervention for students in the randomly assigned quasi-experimental groups—1st grade (waitlist control) and 2nd grade (intervention). Effects were analyzed through repeated quantitative measures of student covitality and EF as dependent variables to determine within- and between-group differences from pretest to posttest. Results from teacher-rated EF and self-reported covitality indicated improvements in both groups over time; however, notable distinctions emerged between groups. 

Within-groups analysis of mean differences indicated significantly greater improvement in EF for the intervention group compared to the waitlist control group. This finding was supported through a statistically significant correlation that emerged between assessment of EF and group condition (*r* = −0.54, *p* = 0.006) at posttest, in which lower BRIEF-2 [63] scores demonstrated greater improvements. Further, intervention group mean GEC scores (BRIEF-2) [63] reflected a favorable shift in EF clinical classification from potentially clinically elevated to no clinical elevation. Self-reported covitality scores also increased from pretest to posttest and demonstrated additional improvements through a shift in survey strengths classification from high-average to high, although results were statistically nonsignificant. 

Similarly, results from within-groups analysis of mean differences for the waitlist control group indicated statistically significant improvement in teacher-rated EF between pretest and posttest, at which mean GEC scores (BRIEF-2) [63] reflected a favorable shift in EF clinical classification from mildly elevated to potentially clinically elevated. Further, self-reported covitality scores increased from pretest to posttest; however, results did not indicate sufficient improvement to cause a shift in survey strengths classification, nor were they found to be statistically significant. Again, we urge caution in interpreting covitality findings for the waitlist control group due to violations of normality of data at posttest; we do, however, provide the analyzed data in the spirit of transparency and to advance future scholarship in the field.

## 4. Discussion

Students’ experiences in school are shaped by constant interactions among cognitive, affective, social, and behavioral domains as they occur within a dynamic system. For instance, the ecological systems theory of development [72] presents a logical framework to explain how children may interpret experiences pivotal to their wellbeing based on context, with school being one such environment. Prior research has provided evidence of contextualized differences between school-based and global SWB correlates, emphasizing the significant implications of how the construct is conceptualized, measured, and utilized to drive interventions in school [3]. 

Despite abundant evidence demonstrating the value of studying and attending to one’s psychological wellness as a conduit for promoting healthy cognitive development, emotional adaptation, positive personality traits, and successful interpersonal relationships for a range of populations across various contexts [6,7], SWB in early childhood education for ND students has remained largely unexplored. Consequently, a distinct need has emerged for greater focus on school-based SWB with diverse student populations, relative to their unique educational experiences and contexts, to add to the limited, but promising, research. Thus, this pilot study was designed to examine practical evaluation and intervention strategies that provide opportunities to foster SBWB through contextualized, collaborative efforts within the systemic socioecological school environment. 

Repeated evaluations of teacher-rated EF and student-reported covitality, before and after participation in an intervention or waitlist control condition, produced promising evidence to support (a) the effects of the *Student Strengths Safari* on covitality and EF for young children, and (b) the utility of a new theoretical dual-factor model to advance SBWB in the service of student neurodiversity in early elementary education. 

### 4.1. Intervention Implications

As a supplement to the schoolwide social learning curriculum delivered in the partner school setting, the covitality intervention implemented in this study may have mitigated behavioral challenges commonly associated with ND. Students in the intervention group demonstrated statistically and clinically significant improvements in teacher-rated EF when compared to their waitlist control group peers over time. Although the small sample size and quasi-experimental design lead us to be cautious about overinterpreting this finding, it is a promising one that is ripe for additional research.

#### 4.1.1. Covitality

Although statistically nonsignificant, covitality scores increased over time within each group, and between-group distinctions emerged. The intervention group reported categorical improvement in their overall SEHS strengths classification [19], whereas levels of SEHS strengths reported by the waitlist control group remained stable. That is, despite identical covitality scores at pretest, the intervention group fared better than their waitlist control group peers over time, illustrated by greater improvement in levels of student covitality indicated by a group mean score which fell within a new classification range of high SEHS strengths. By contrast, increased levels of student covitality reported by the waitlist control group were insufficient to cross the threshold into a new range of SEHS strengths group classification. This finding indicates that although covitality scores reported by the intervention group were not statistically significant, they are practically significant in driving socially valid intervention and evaluation efforts in real-world settings.

Small sample sizes are a recognized limitation to demonstrating statistical significance in school-situated research due to the lack of statistical power that is often necessary to show such an effect. However, these sample sizes reflect the classroom characteristics that professionals in the field encounter and provide ecological validity to support the results of this study. Furthermore, the SEHS-P [19] has not yet been validated as a progress monitoring tool that is sensitive to change over time; therefore, it may not be sensitive enough to detect small to moderate intervention effects. For instance, although the intervention group reported high SEHS strengths at posttest, the average score fell within 3-points of the control group, which remained in the high-average SEHS strengths group classification. Thus, more research is needed to validate the tool as a sensitive measurement to indicate change over time.

#### 4.1.2. Executive Functioning

On the other hand, statistically significant improvements and corresponding shifts in clinical classification profiles of EF were recognized within each group over time. Clinical classification of risk levels in the intervention group shifted from potentially clinically elevated to no clinical elevation, and the waitlist control group shifted from mildly elevated to potentially clinically elevated. These shifts contributed new insight toward a revised classification system derived from the new dual-factor intervention framework, whereby holistic student profiles are identified at the intersection of covitality and EF through comprehensive evaluation of SBWB.

Of particular note, however, was the statistically significant interaction effect which indicated observable improvements in EF for the intervention group that were significantly greater than the gains made over time in the waitlist control group. Furthermore, this outcome was corroborated by a significant correlation linking EF to group condition at posttest. Taken together, these results indicate preliminary evidence to support the covitality model as a meaningful framework for guiding school-based screening, progress monitoring, and intervention efforts in early elementary education that are not only evidence-based but are socially valid and adaptable for practical infusion into the school environment.

### 4.2. Multidimensional Student Profiles

Moreover, multidimensional outcomes provided evidence to support the *Student Strengths Safari* as an effective intervention to augment the schoolwide integrated social skills curriculum. Consistent with a dual-factor framework, that students’ observed positive behaviors—or the perceived absence of behavioral challenges—were insufficient to stand alone as the sole indicator of SBWB. Although valuable in its own right, high EF did not equate to high covitality, as these factors represented distinct, but interrelated constructs that together advance understanding of SBWB for young ND students.

Social skills training and behavioral intervention programming are commonly introduced into standard educational practice to address the challenges students with social cognition differences encounter in school settings. However, this study provided new evidence of the variance among students with wide-ranging executive functioning skill sets and student strength profiles. Together, these skills and strengths provide a more comprehensive picture of SBWB illustrated through four student profiles derived from Keyes’s [12] dual-factor model and adapted for this unique student population: thriving, content, vulnerable, and languishing. These profiles offer a roadmap to new pathways in which education professionals can support student success through fine-tuned methods of evaluation and intervention that are both developmentally appropriate and effective. For example, using this new model of SBWB for young ND students, school personnel may be better able to tailor targeted interventions that capitalize on students’ strengths and address their needs through a holistic approach to education and development.

### 4.3. Summary of Intervention Implications

Neurological differences that impact students’ social-emotional health and EF can present challenges both for learning and the development of meaningful interpersonal relationships in school. Strong relationships and requisite adaptive social learning and behaviors can serve as protective factors to mitigate distress caused by a mismatch between environmental features and available resources to successfully maneuver in the world [73]. Because neurodiversity can manifest in a variety of ways and exacerbate environmental stressors on psychological wellbeing, and because early childhood education provides an ideal time and place for developmentally appropriate and ecologically valid intervention, multidimensional student profiles constructed through the novel dual-factor model of SBWB explored in this study can inform comprehensive intervention strategies. These may include combined foci on cognitive skill sets and social-emotional strengths that honor students’ unique experiences of neurodivergence. These profiles may be used to inform targeted interventions that accommodate the specific needs of each student group reflected in this model. Children are especially prone to internalizing negative experiences that adversely impact development and functioning; thus, it is crucial for caretakers, educators, and service providers to identify effective strategies that both mitigate the negative effects of stressors and optimize academic, social, psychological and emotional wellbeing across the lifespan. 

### 4.4. Recommendations for Research, Policy, and Practice

#### 4.4.1. Multidisciplinary Collaboration in Multidimensional Education

The multidimensional view of health set forth by the World Health Organization [74] has advanced understandings of educational wellness through the promotion of SWB with the recent use of CMH screening in schools [64]. Unfortunately, a dual-factor approach to SBWB continues to be overlooked as a priority in the current political climate, which largely emphasizes academic achievement through objective, standardized measures. However, findings from this study indicated that higher student covitality was linked to improved teacher-rated of EF for young students following participation in a novel covitality intervention. This evidence can inform progressive policy endeavors that promote a framework for instruction which places equal emphasis on social-emotional health and executive functioning to foster optimal outcomes in a developmentally appropriate learning environment, beginning at the earliest stages of educational instruction. 

Further, this study is the first investigation of student covitality in a population that represents two demographic groups which had not previously been represented in the research literature: (a) students in early childhood education (1st and 2nd grade), and (b) students who are neurodivergent. The second-order latent construct of student covitality is the first model that has been tested and validated in prior research with older elementary education students to inform SBWB; therefore, there was value in analyzing the four underlying factors as dependent variables for insight into whether they are meaningful for younger students representing the neurodiversity population.

Multidisciplinary teams of researchers, educators, administrators, and mental health professionals possess invaluable cumulative expertise necessary to evaluate and drive wellbeing prevention and intervention strategies that reflect the multidimensionality of student covitality. Due to the unique nature of SWB as an individualized psychological determinant of health which varies in meaning across contexts [3], multidisciplinary collaboration must be regarded as a priority to wellbeing promotion. In response, future policy initiatives must align with person-centered priorities to advance SBWB by serving the whole student through holistic and collaborative professional efforts.

The current investigation was conducted in partnership with a small, specialized, private pay school, in an affluent Mid-Atlantic suburb. Further replication studies are needed to gain a more comprehensive view of covitality for diverse students to ensure that we, as a collective field, are meeting all students where they are. Future research should include explorations of covitality with students across contexts, such as school setting, geographical region, socioeconomic status, race, ethnicity, and other demographic variables of interest that can provide insight into how we can best cultivate environments of belonging that foster positive identity development.

Finally, although both groups’ scores improved over time on quantitative assessments, the intervention group showed greater categorical improvement in multidimensional group classifications. In a small sample with low statistical power, the visual inspection of dual-factor scatter plots provided important context for additional consideration of potentially practically significant effects that are observable at the teacher-rater level but not in quantitative analyses. As researchers aim to provide ecologically valid interventions and evaluation data that are relevant to school personnel, richly informed conceptual framing and analyses can be valuable approaches to support use-oriented educational research and promising, innovative practice. This will be an important consideration for future research design, implementation, and analysis.

#### 4.4.2. *Student Strengths Safari* Program Replication, Generalization, and Adaptation

Future investigations are needed to replicate the present study with larger sample sizes to enhance rigor through more robust evidentiary support. Further studies should explore the SEHS-P [19] measurement tool’s sensitivity to change for consistent progress monitoring and consider replications and adaptations to data collection and intervention frequency, dosage, and time. Due to the brevity of the current study as an 8-session intervention over four consecutive weeks, with limited data collected and evaluated across two time points at pretest and posttest, future investigations should include additional measurement across additional timepoints to examine intervention maintenance, including longitudinal stability of the multidimensional student profiles developed through the new dual-factor model of SBWB. Delayed posttest data and more complex longitudinal investigations are also needed to better understand the stability of intervention effects on student covitality and EF over time. Such exploration is needed to provide more insight into potential practices that may be implemented or adapted to improve intervention outcomes and sustain positive effects over time.

Furthermore, future considerations should include intervention adaptations for culturally and developmentally appropriate expansion to determine the utility of the *Student Strengths Safari* program to drive ecologically valid educational advancement for diverse student groups across grade levels, educational settings, and cultural contexts. For example, how would intervention outcomes differ among older ND elementary students, or cross-cultural student groups with whom the SEHS-P [19] has been validated in prior research? Additionally, would intervention efficacy change as a function of various programmatic themes (e.g., Student Strengths “Road Trip,” or “Desert Excursion,” etc.), or would particular themes appeal to certain student demographics more so than others? 

The present study provided evidence of the covitality intervention as beneficial for early elementary students who are neurodivergent; however, increasing interest in using the covitality model for targeted interventions has emerged across the U.S. and around the world, through ongoing investigations by the UC Santa Barbara Project Covitality research team, and affiliates in Australia, Slovakia, Italy, England, Indonesia, and Japan [75]. This steady progress toward international expansion of the SEHS System—the SEHS-P [19] alone has been adapted linguistically and validated for use in Turkish [76], Chinese [69], Korean [77], and Spanish [78] cultures—is driving widespread concentration on developmental initiatives for practical strategies that foster student covitality strengths across educational contexts.

Future adaptations of the Student Strengths Safari program can target the expanded survey strengths measured among students in secondary and higher education. Screening and intervention procedures for these student groups should include implementation of the SEHS-S/HE [19,79] in conjunction with program components inclusive of developmentally adapted gratitude, optimism, zest, and persistence strategies outlined in this study, as well as evidence-based practices that address the additional student strengths of self-awareness, self-efficacy, school support, family coherence, peer support, empathy, self-control, and emotion regulation that are unique to the SEHS-S/HE [19,79]. In addition to the necessary replications, the proposed investigative expansions allow for more nuanced deliberations in determining longevity for advancing social-emotional health and executive functioning through a unique dual-factor model of SBWB, and further enhance the generalizability of the Student Strengths Safari intervention program through ongoing discovery and practical integration of flexible designs that conform to diverse socioecological educational environments.

### 4.5. Limitations 

There are a number of limitations in this study, and we encourage cautious interpretation of study findings. The small sample size and quasi-experimental design limit the ability to draw confident inferences about quantitative results. In applied, school-situated research with young children, small, nested samples and quasi-experimental designs are ubiquitous, but make it challenging to confidently attribute between-group differences at posttest to the covitality intervention. Furthermore, convenience sampling limited the availability of a robust sample size and diminished statistical power needed to detect any small to moderate effects of the intervention on the covitality scale. Time constraints limited the intervention to 4 weeks between pretest and posttest measures; thus, reducing the threat of within-group maturation and increasing the likelihood that significant improvements found over time within the intervention group, relative to the control group, may be attributed to effects of the intervention. On the other hand, the waitlist control group was one year younger than the intervention group, and we cannot be certain that observed mean differences between groups over time are not due to naturally occurring development in how 1st and 2nd graders “settle into” school and improve their executive functioning behaviors in response to school expectations and routines. As this study was also conducted at one independent school, findings may not be generalizable. 

It is also important to note once more that the research partnership school is a small, specialized, private-pay educational institution. The tools and resources that were most appropriate and recommended by teachers to enhance intervention implementation may not be readily available and accessible to other school environments. Furthermore, the population at this school reflects a range of student neurodiversity, and, for some, the abstract concepts (e.g., gratitude) were difficult to grasp; therefore, it is possible that response bias, particularly acquiescence bias and social desirability bias, may have influenced student responses on self-reported survey items. 

## 5. Conclusions

Early childhood represents a period of heightened sensitivity in which environmental factors can have lasting influences on neurological development that may not otherwise develop from similar experiences later in life [72,80]. Findings presented in this study emphasized specific strengths and skills (i.e., social-emotional and executive functioning) as important objectives for future early education interventions to promote optimal student development.

The paucity of research available regarding strengths-oriented practices with young ND students may be attributable to anticipated challenges associated with developmental and/or cognitive maturity. However, this population represents one in particular that can benefit significantly from strengths-focused early intervention, as practices are grounded in the facilitation of social, emotional, cognitive, and behavioral health—skills that are often targeted through social or behavioral interventions. A holistic snapshot of students’ transactional experiences within the education environment must capture the interconnectivity of these domains and acknowledge and account for the multidimensionality of psychological processing that may not have been captured from unidimensional evaluation in school-based contexts thus far. Furthermore, educational instruction to support healthy adaptation in the early years should be introduced at pivotal learning stages when children begin to internalize experiences that shape their worldviews and construct foundational dispositions that influence ongoing identity development.

This investigation provided initial evidence of young neurodivergent students’ capacity to flourish in education environments that attend to both universal strengths and targeted skills through multicomponent instruction. Above all, the resulting dual-factor model of SBWB that emerged from this cross-disciplinary collaboration provides a pathway to jumpstart the social-emotional development of children who often encounter challenges in school. Finally, covitality interventions, such as the Student Strengths Safari, can be leveraged to support students as individuals, or as a collective group in cultivating identities rich with purpose, passion, and unlimited potential to thrive in school and life.

## Figures and Tables

**Table 1:** Descriptive statistics of covitality and executive functioning measured over time.

Measure	Mean		Median		Standard Deviation		Range	
	Pre	Post	Pre	Post	Pre	Post	Pre	Post
**Total CoVi**	52.4	55.4	55.5	59.5	9.88	9.10	34–64	28–64
**Gratitude**	13.9	14.5	14	16	2.21	2.04	10–16	10–16
**Optimism**	13.3	13.5	14	14	2.94	2.69	6–16	8–16
**Zest**	12.7	13.8	14	15	3.09	2.97	6–16	4–16
**Persistence**	12.5	13.5	13	14	3.46	2.74	4–16	6–16
**EF (GEC)**	68.4	59.5	68.5	59.0	10.6	9.63	46–91	44–84

Note. *n* = 24; Total CoVi = covitality summed score, scale of up to 64; Covitality subscales, scale of 4–16; EF = executive functioning; GEC = global executive composite, standardized scale (*M* = 50, *SD* = 10).

**Table 2:** Tests of within-groups contrasts measured over time.

Source	Measure	Time	Type III Sum of Squares	df	Mean Square	*F*	Sig.	Partial Eta Squared
**time**	EF	Post vs. Pre	2080.576	1	2080.576	69.6	0.000	0.760
	CoVi	Post vs. Pre	243.219	1	243.219	4.10	0.055	0.157
**time × condition**	EF	Post vs. Pre	232.576	1	232.576	7.79	0.011	0.261
	CoVi	Post vs. Pre	43.886	1	43.886	0.739	0.399	0.033
**Error** **(time)**	EF	Post vs. Pre	657.257	22	29.875			
	CoVi	Post vs. Pre	1305.614	22	59.346			

Note. EF = executive functioning; CoVi = covitality.

**Table 3:** Tests of between-groups effects measured over time.

Source	Measure	Type III Sum of Squares	df	Mean Square	*F*	Sig.	Partial Eta Squared
**Intercept**	EF	93,536.305	1	93,536.305	1126	0.000	0.981
	CoVi	68,022.001	1	68,022.001	867	0.000	0.975
**Condition**	EF	304.805	1	304.805	3.67	0.069	0.143
	CoVi	11.668	1	11.668	0.149	0.703	0.007
**Error**	EF	1827.529	22	83.069			
	CoVi	1725.832	22	78.447			

Note. EF = executive functioning; CoVi = covitality.

**Table 4:** Contrast Results (K Matrix).

Group Difference Contrast			Averaged Variable	
			EF	CoVi *
Posttest vs. Pretest	Contrast Estimate		−7.229	1.414
	Hypothesized Value		0	0
	Difference (Estimate-Hypothesized)		−7.229	1.414
	Std. Error		3.774	3.667
	Sig.		0.069	0.703
	95% Confidence Interval for Difference	Lower Bound	−15.055	−6.191
		Upper Bound	0.598	9.02

Note. EF = executive functioning; CoVi = covitality. * Cautious interpretation is warranted due to non-normality of data for the waitlist control group at posttest.

**Table 5:** Estimated marginal means—grand mean.

Measure	Mean	Std. Error	95% Confidence Interval	
			Lower Bound	Upper Bound
EF	63.314	1.887	59.401	67.227
CoVi	53.993	1.834	50.19	57.795

Note. EF = executive functioning; CoVi = covitality.

**Table 6:** Multidimensional group profiles measured over time.

Measurement Tool Classification System	Group	Category	
		Pretest	Posttest
**BRIEF-2 Risk**	Grade 1 (Control)	Clinically elevated	Mildly elevated
	Grade 2 (Intervention)	Potentially clinically elevated	No clinical elevation
**SEHS Strengths**	Grade 1 (Control)	High-average	High-average
	Grade 2 (Intervention)	High-average	High

Note. Student strength/risk profiles categorized by measurement tool classification systems assessed over time; SEHS = Social Emotional Health Survey [19]; BRIEF-2 = Behavior Rating Inventory of Executive Function^®^, Second Edition [63].

## Data Availability

The data presented in this study are available on request from the corresponding author.

## References

[1] Furlong M.J., Dowdy E., Nylund-Gibson K., Wagle R., Carter D., Hinton T. (2021). Enhancement and standardization of a universal social-emotional health measure for students’ psychological strengths. J. Well-Being Assess..

[2] Diener E. (2000). Subjective well-being. The science of happiness and a proposal for a happiness index. Am. Psychol..

[3] Long R.F., Huebner E.S., Wedell D.H., Hills K.J. (2012). Measuring school-related subjective well-being in adolescents. Am. J. Orthopsychiatr..

[4] Keyes C.L.M. (2007). Promoting and protecting mental health as flourishing: A complimentary strategy for improving national mental health. Am. Psychol..

[5] Ryan R.M., Deci E.L. (2001). On happiness and human potentials: A review of research on hedonic and eudaimonic well-being. Annu Rev. Psychol..

[6] Lyubomirsky S., King L., Diener E. (2005). The benefits of frequent positive affect: Does happiness lead to success?. Psychol. Bull..

[7] Cohen S., Pressman S.D. (2006). Positive affect and health. Curr. Dir. Psychol. Sci..

[8] Keyes C.L.M. (2006). Mental health in adolescence: Is America’s youth flourishing?. Am. J. Orthopsychiatr..

[9] Park N. (2004). The role of subjective well-being in positive youth development. An. Am. Acad. Pol. Soc. Sci..

[10] Greenspoon P.J., Saklofske D.H. (2001). Toward an integration of subjective well-being and psychopathology. Soc. Indic. Res..

[11] Suldo S.M., Shaffer E.J. (2008). Looking beyond psychopathology: The dual factor model of mental health in youth. Sch. Psych. Rev..

[12] Keyes C.L.M. (2005). Mental illness or mental health? Investigating axioms of the complete state model of mental health. J. Consult. Clin. Psychol..

[13] Keyes C.L.M., Shmotkin D., Ryff C.D. (2002). Optimizing well-being: The empirical encounter of two traditions. J. Pers. Soc. Psychol..

[14] American Psychiatric Association (2000). Diagnostic and Statistical Manual of Mental Disorders.

[15] Antaramian S.P., Huebner E.S., Hills K.J., Valois R.F. (2010). A dual-factor model of mental health: Toward a more comprehensive understanding of youth functioning. Am. J. Orthopsychiatr..

[16] Suldo S.M., Talji-Raitano A., Kiefer S.M., Ferron J.M. (2016). Conceptualizing high school students’ mental health through a dual factor model. Sch. Psych. Rev..

[17] Eklund K., Dowdy E., Jones C., Furlong M.J. (2010). Applicability of the dual factor model of mental health for college students. J. Coll. Stud. Psychother..

[18] Renshaw T.L., Cohen A.S. (2014). Life satisfaction as a distinguishing indicator of college student functioning: Further validation of the two-continua model of mental health. Soc. Indic. Res..

[19] Furlong M.J., You S., Renshaw T.L., O’Malley M.D., Rebelez J. (2013). Preliminary development of the Positive Experiences at School Scale for elementary school children. Child. Indic. Res..

[20] Ghielen S.T.S., van Woerkom M., Meyers M.C. (2017). Promoting positive outcomes through strengths interventions: A literature review. J. Posit. Psychol..

[21] Division for Early Childhood Position Statement. www.decdocs.org/brief-personnel-standards.

[22] Robison J.E. What Is Neurodiversity?. www.psychologytoday.com/us/blog/my-life-aspergers/201310/what-is-neurodiversity.

[23] Baglieri S., Valle J.W., Connor D.J., Gallagher D.J. (2011). Disability studies in education: The need for a plurality of perspectives on disability. Remedial Spec. Educ..

[24] Lovaas O.I., Koegel R., Simmons J.Q., Long J.S. (1973). Some generalizations and follow-up measures on autistic children in behavior therapy. J. Appl. Behav. Anal..

[25] Matson J.L., Matson M.L., Rivet T.T. (2007). Social skills treatments for children with autism spectrum disorders: An overview. Behav. Modif..

[26] Center for Child Well-Being Strengths-Based vs. Deficit-Based Approaches. www.fromhungertohealth.files.wordpress.com/2016/02/strengthsvsdeficitrb.pdf.

[27] Harry B., Klingner J. (2007). Discarding the deficit model. Educ. Leadersh..

[28] Dinashak J. (2016). The deficit view and its critics. Disabil. Stud. Q..

[29] Wehmeyer M.L., Buntix W.H.E., Lachapelle Y., Luckasson R.A., Schalock R.L., Verdugo M.A., Borthwick-Duffy S., Bradley V., Craig E.M., Coulter D.L. (2008). The intellectual disability construct and its relation to human functioning. Intell. Dev. Disabil..

[30] Seligman M.E.P., Ernst R.M., Gillham J., Reivich K., Linkins M. (2009). Positive education: Positive psychology and classroom interventions. Oxf. Rev. Educ..

[31] Sin N.L., Lyubomirsky S. (2009). Enhancing well-being and alleviating depressive symptoms with positive psychology interventions. A practice-friendly meta-analysis. J. Clin. Psychol..

[32] Gilman R., Huebner E.S., Furlong M.J. (2014). Handbook of Positive Psychology in Schools.

[33] Quinlan D.M., Swain N., Cameron C., Vella-Brodrick D.A. (2015). How ‘other people matter’ in a classroom-based strengths intervention: Exploring interpersonal strategies and classroom outcomes. J. Posit. Psychol..

[34] Shoshani A., Steinmetz S., Kanat-Maymon Y. (2016). Effects of the Maytiv positive psychology school program on early adolescents’ mental health and well-being. J. Happiness Stud..

[35] Suldo S.M., Hearon B.V., Bander B., McCullough M., Garofano J., Roth R., Tan S. (2015). Increasing elementary school students’ subjective well-being through a classwide positive psychology intervention: Results of a pilot study. Contemp. Sch. Psychol..

[36] Owens R.L., Patterson M.M. (2013). Positive psychological interventions for children: A comparison of gratitude and best possible selves approaches. J. Genet. Psyschol..

[37] Niemiec R.M., Shogren K.A., Wehmeyer M.L. (2017). Character strengths and intellectual and developmental disability: A strengths-based approach from positive psychology. Educ. Train. Autism Dev. Disabil..

[38] Seligman M.E.P., Csikszentmihalyi M. (2000). Positive psychology: An introduction. Am. Psychol..

[39] Peterson C., Seligman M.E.P. (2004). Character Strengths and Virtues: A Handbook and Classification.

[40] Armstrong K.H., Missall K.N., Schaffer E.I., Hoinoski R.L., Gilman R., Huebner E.S., Furlong M.J. (2009). Promoting positive adaptation during the early childhood years. Handbook of Positive Psychology in Schools.

[41] Niemiec R.M. (2014). Mindfulness and Character Strengths: A Practical Guide to Flourishing.

[42] Biswas-Diener R. (2006). From the equator to the North Pole: A study of character strengths. J. Happiness Stud..

[43] Dahlsgaard K., Peterson C., Seligman M.E.P. (2005). Shared virtue: The convergence of valued human strengths across culture and history. Rev. Gen. Psychol..

[44] Ladd G.W. (1990). Having friends, keeping friends, making friends, and being liked by peers in the classroom: Predictors of children’s early school adjustment. Child. Dev..

[45] Ladd G.W., Coleman C.C. (1997). Children’s classroom peer relationships and early school attitudes: Concurrent and longitudinal associations. Early Educ. Dev..

[46] Ladd G.W., Birch S.H., Buhs E.S. (1999). Children’s social and scholastic lives in kindergarten: Related spheres of influence?. Child. Dev..

[47] McClelland M.M., Morrison F.J., Holmes D.L. (2000). Children at risk for early academic problems: The role of learning-related social skills. Early Child. Res. Q..

[48] Park N., Peterson C. (2006). Character strengths and happiness among young children: Content analysis of parental descriptions. J. Happiness Stud..

[49] Park N., Peterson C. (2006). Moral competence and character strengths among adolescents: The development and validation of the Values in Action Inventory of Strengths for Youth. J. Adolesc..

[50] Hay D.F., Castle J., Stimson C.A., Davies L., Killen M., Hart D. (1999). The social construction of character in toddlerhood. Morality in Everyday Life: Developmental Perspectives.

[51] Weiss A., King J.E., Enns R.M. (2002). Subjective well-being is heritable and genetically correlated with dominance in chimpanzees (*pan troglodytes*). J. Pers. Soc. Psychol..

[52] Wilkins B., Boman P., Mergler A. (2015). Positive psychological strengths and school engagement in primary school children. Cogent Educ..

[53] Lee S., You S., Furlong M.J. (2015). Validation of the social emotional health survey for Korean school students. Child. Indic. Res..

[54] Kim E.K., Furlong M.J., Dowdy E., Felix E.D. (2014). Exploring the relative contribution of the strength and distress components of dual-factor complete mental health screening. Can. J. Sch. Psychol..

[55] Furlong M.J., You S., Renshaw T.L., Smith D.C., O’Malley M.D. (2014). Preliminary development and validation of the Social and Emotional Health Survey for secondary students. Soc. Indic. Res..

[56] Hearon B.V. (2017). Promoting Happiness in Elementary Schoolchildren: Evaluation of a Multitarget, Multicomponent Classwide Positive Psychology Intervention. Ph.D. Thesis.

[57] Rashid T., Anjum A., Lennox C., Quinlan D., Niemiec R.M., Mayerson D., Kazemi F., Proctor C., Linley P.A. (2013). Assessment of character strengths in children and adolescents. Research, Applications, and Interventions for Children and Adolescents: A Positive Psychology Perspective.

[58] Shoshani A., Steinmetz S. (2014). Positive psychology at school: A school-based intervention to promote adolescents’ mental health and well-being. J. Happiness Stud..

[59] Suldo S.M., Savage J.A., Mercer S.H. (2014). Increasing middle school students’ life satisfaction: Efficacy of a positive psychology group intervention. J. Happiness Stud..

[60] Roth R., Suldo S.M., Ferron J. (2017). Improving middle school students’ subjective well-being: Efficacy of a multi-component positive psychology intervention targeting small groups of youth and parents. Sch. Psych. Rev..

[61] Wingate E.J., Suldo S.M., Peterson R.K. (2018). Monitoring and fostering elementary school students’ life satisfaction: A case study. J. Appl. Sch. Psychol..

[62] Naples L.H. (2019). Neurodivergence in Early Childhood: Deriving a Dual-Factor Model of Educational Well-Being Through a Design-Based Research Pilot Program. Ed.D. Thesis.

[63] Gioia G.A., Isquith P.K., Guy S.C., Kenworthy L. (2016). Behavior Rating Inventory of Executive Function (BRIEF-2).

[64] Dowdy E., Furlong M.J., Raines T., Bovery B., Kauffman B., Kamphaus R.W., Dever B.V., Price M., Murdock J. (2015). Enhancing school-based mental health services with a preventive and promotive approach to universal screening for complete mental health. J. Educ. Psychol. Consult..

[65] Creswell J.W., Plano Clark V.L. (2018). Designing and Conducting Mixed Methods Research.

[66] Haggerty P. Five Private School Federal Aid Myths Debunked. www.factsmgt.com/blog/5-private-school-federal-aid-myths-debunked/.

[67] Broughman S.P., Rettig A., Peterson J. (2017). Characteristics of Private Schools in the United States: Results from the 2015–2016 Private School Universe Survey.

[68] Renshaw T.L. (2017). Technical adequacy of the Positive Experiences at School Scale with adolescents. J. Psychoeduc. Assess..

[69] Wang C., Yang C., Jiang X., Furlong M.J. (2016). Validation of the Chinese version of the Social Emotional Health Survey-Primary. Int. J. Sch. Educ. Psychol..

[70] Gioia G.A., Isquith P.K., Roth R.M., Kreutzer J.S., DeLuca J., Caplan B. (2018). Behavior rating inventory for executive function. Encyclopedia of Clinical Neuropsychology.

[71] Shogren K.A., Wehmeyer M.L., Forber-Pratt A.J., Palmer S.B. (2015). VIA Inventory of Strengths for Youth (VIA-Youth): Supplement for Use When Supporting Youth with Intellectual and Developmental Disabilities to Complete the VIA-Youth.

[72] Bronfenbrenner U. (1979). The Ecology of Human Development.

[73] Shogren K.A., Wehmeyer M.L. (2013). Positive psychology and disability: A historical analysis. The Oxford Handbook of Positive Psychology and Disability.

[74] Candau M.G., World Health Organization (1965). The Work of WHO, 1964: Annual Report of the Director-General to the World Health Assembly and to the United Nations.

[75] Social Emotional Health Survey System Publications Project Covitality: A School Mental Wellness and Thriving Student Development Initiative Gervitz Graduate School of Education International Center for School Based Youth Development Social Emotional Health Survey System. https://www.researchgate.net/publication/330335372_Social_Emotional_Health_Survey_System_Publications_Project_Covitality_A_school_mental_wellness_and_thriving_student_development_initiative_Gevirtz_Graduate_School_of_Education_International_Center_for.

[76] Telef B.B. (2016). Validity and reliability study of Positive Experiences at School Scale 2016 (Okulda Pozitif Yaşantılar Ölçeği geçerlik ve güvenirlik çalışması). J. Hum. Sci. (Uluslar. İnsan Bilim. Derg.).

[77] Kim E., Dowdy E., Furlong M.J., You S. (2018). Complete mental health screening: Psychological strengths and life satisfaction in Korean students. Child. Indic. Res..

[78] Pineda D., Piqueras J.A., Martinez A., Rodriguez-Jimenez T., Martínez Gonzalez A.E., Santamaria P., Furlong M.J. A New Instrument for Covitality: The Revised Social Emotional Health Survey-Primary in a Spanish Sample of Children. Proceedings of the 14th European Conference on Psychological Assessment.

[79] Furlong M.J., You S., Shishim M., Dowdy E. (2017). Development and validation of the Social Emotional Health Survey—Higher Education version. Appl. Res. Qual. Life.

[80] Shonkoff J.P., Phillips D.A. (2000). From Neurons to Neighborhoods: The Science of Early Childhood Development.

